# Amine Responsive Poly(lactic acid) (PLA) and Succinic Anhydride (SAh) Graft-Polymer: Synthesis and Characterization

**DOI:** 10.3390/polym11091466

**Published:** 2019-09-07

**Authors:** Adrián Lopera-Valle, Anastasia Elias

**Affiliations:** Department of Chemical and Materials Engineering, University of Alberta, Donadeo Innovation Centre for Engineering, Edmonton, AB T6G 1H9, Canada; lopera@ualberta.ca

**Keywords:** amine sensing, free radical polymerization (FRP), maleic anhydride, methylamine, poly(lactic acid), succinic anhydride

## Abstract

Amines are known to react with succinic anhydride (SAh), which in reactions near room temperature, undergoes a ring opening amidation reaction to form succinamic acid (succinic acid-amine). In this work, we propose to form an amine-responsive polymer by grafting SAh to a poly(lactic acid) (PLA) backbone, such that the PLA can provide chemical and mechanical stability for the functional SAh during the amidation reaction. Grafting is performed in a toluene solution at mass content from 10 wt% to 75 wt% maleic anhydride (MAh) (with respect to PLA and initiator), and films are then cast. The molecular weight and thermal properties of the various grafted polymers are measured by gel permeation chromatography and differential scanning calorimetry, and the chemical modification of these materials is examined using infrared spectroscopy. The efficiency of the grafting reaction is estimated with thermogravimetric analysis. The degree of grafting is determined to range from 5% to 42%; this high degree of grafting is desirable to engineer an amine-responsive material. The response of the graft-polymers to amines is characterized using X-ray photoelectron spectroscopy, infrared spectroscopy, and differential scanning calorimetry. Changes in the chemical and thermal properties of the graft-polymers are observed after exposure to the vapors from a 400 ppm methylamine solution. In contrast to these changes, control samples of neat PLA do not undergo comparable changes in properties upon exposure to methylamine vapor. In addition, the PLA-g-SAh do not undergo changes in structure when exposed to vapors from deionized water without amines. This work presents potential opportunities for the development of real-time amine sensors.

## 1. Introduction

Sensors based on smart materials, which undergo visible and gradual changes in color in response to a stimulus of interest, can be used effectively to monitor both storage conditions and analytes indicative of food safety. For example, time-temperature indicators, which undergo gradual changes in color at a temperature-dependent rate, can reliably track the refrigeration history of goods, which is vital for food preservation [1]. While storage conditions such as temperature, humidity, and oxygen are indirect indicators of freshness, it is also desirable to monitor direct analytes such as chemicals released during food ripening (e.g., ethylene oxide [2]). In the case of meat and fish products, biogenic amines including ammonia, putrescine, dimethylamine, putrescine, dopamine, histamine, and methylamine are known markers of spoilage [3,4,5,6]. The concentration of amines present in an enclosed fish package, for instance, has been shown to increase from 130 ppm to 350 ppm at 4 °C with time as food decreases in freshness [4,7]; gaseous and liquid amines potentially represent direct indicators of food freshness and safety. 

The concentration of amines in solution, gas, or vapor phases can be measured by high performance liquid chromatography [8,9], electrochemical sensors [10,11], electrical-based sensors [12], and optical detection methods [3,13]. Most of these methods require lab-scale analytic equipment and trained personnel. However, smart materials, that undergo a specific and sensitive reaction to a stimulus, can be used for the detection of amines. For instance, Jin et al. proposed the use of nitrated polythiophene (NPTh) for the detection of a wide range of biogenic amines (BAs). In this system, the BAs easily diffuse into the polymer film and form charge transfer complexes with NPTh, leading to a change in the color of the film [14]. A key component of this system is a material that reacts selectively with amines.

Amine-selective reactants that have been incorporated into electrically-responsive smart materials are anhydrides such as maleic anhydride (MAh) and succinic anhydride (SAh). As reported previously, in the first step of the reaction between the amine and MAh or SAh, the lone electron pair of the amine conducts a nucleophilic attack on the C=O π bond of the anhydride to form a tetrahedral intermediate, while the second carbonyl becomes part of the leaving group on the other side of the anhydride ring [15,16,17,18,19,20,21,22]. This is followed by the removal of a proton from the leaving group by the amine and the donation of an electron from the carboxylate group back to the C=O π bond, which is much less electrophilic than in an acid anhydride [15,16,17,18,19,20]. While the reaction between MAh or SAh and amines has been used as initial step for classical approaches to the synthesis of maleimide and succinimide, this process requires the dehydration of the intermediate acid, usually promoted by acids and temperature [21,23,24]. At room temperature, the most likely pathway is that the ring-opening reaction converts the anhydride into the corresponding dicarboxylic acid monoamide, maleamic acid or succinamic acid for MAh and SAh, respectively [15,17,21]. As reviewed by Sun et al. [25], polymers that undergo molecular structural changes—in this case the ring opening of SAh upon contact with methylamine—have high potential as stimuli responsive materials. Such transitions could potentially be leveraged in smart material-based systems, using a pH indicator dye, as the acidity of the anhydride changes, or using an electrochemical set up to monitor the creation of H+ ions in the anhydride–amine reaction, or changes in the water solubility of the compound, as MAh and SAh are not soluble in water while maleamic and succinamic acids are.

**Figure 1:**
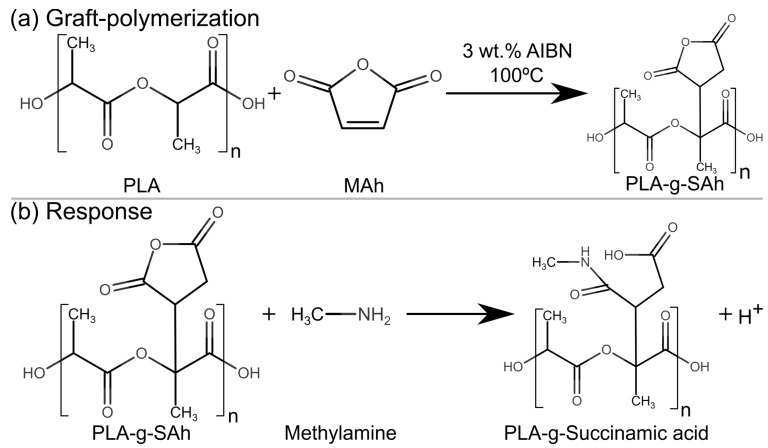
Proposed free radical graft-polymerization reaction between poly(lactic acid) (PLA) and maleic anhydride (MAh), and reaction between PLA-*g*-SAh (succinic anhydride) and methylamine to form PLA-*g*-succinamic acid.

In order to engineer amine-responsive materials based on succinic anhydride, chemically-stable materials compatible with food packaging must be produced. In the literature, a number of thermal processing methods have been demonstrated for grafting SAh onto various polymer chains, including polystyrene [19,26], poly(N-isopropyl acrylamide) (NIPA) [27], and polyethylene glycol [28]. One polymer of interest for use in food-based sensors is polylatic acid (PLA). Polylatic acid (PLA, Figure 1a) is a bio-sourced, food compatible, biodegradable, and recyclable thermoplastic with high commercial attention given that, as a biopolymer, it can help mitigate the polymer waste problem, particularly in food packaging [28,29]. One advantage of PLA is that it has functional groups such as –OH and –CH in its chain, which makes possible to chemically couple it with reactive polymers such as maleic and succinic anhydrides [30,31], oxazoline, and epoxide [31,32]. In addition to reacting with amines (as described above), maleic anhydride (MAh) is one of the most widely used reactive compatibilizers in polymer processing due to its good chemical reactivity, low toxicity, and low potential to form dimers, trimers, and polymers under free radical grafting conditions [33,34]. While the in-situ melt graft-polymerization of PLA and MAh, to form PLA-*g*-SAh, by means of free radical polymerization has been studied and reported in the past by Hwang et al. [32], Detyothin et al. [35,36], Ma et al. [37], Du et al. [38], Csikós et al. [39], Birnin-Yauri et al. [40], and others, these methods typically utilized melt-based processes and achieved only very low grafting degree (below 3%). To maximize the response of the materials, a large grafting degree is desired.

In this work, we develop a solution-based method for the synthesis of responsive polymers with SAh in their structure and characterize the response of these materials to a fish degradation biogenic amine: methylamine. Firstly, MAh is grafted with poly(lactic acid) to form PLA-*g*-SAh (Figure 1). In this system, the PLA acts as a backbone/scaffold for the responsive component of material (i.e., the succinic anhydride) (Figure 1a). The method proposed in this work aims to achieve a high grafting rate of SAh to PLA chains. We then study the effect of initial MAh content in the graft-polymer on the physical properties, including molecular weight, polydispersity index, thermal stability, and thermal properties. In order to monitor and prove the responsive behavior of SAh in the graft-polymers, samples are exposed to the vapors from a 400 ppm methylamine solution in water under room conditions (Figure 1b). Following exposure to amines, Fourier-transform infrared spectroscopy (FTIR) and X-ray photoelectron spectroscopy (XPS) are used to confirm and characterize the reaction between amines and SAh. In addition, the thermal properties of the polymer were compared with those of the films’ prior amine exposure.

## 2. Experimental Methods

### 2.1. Polymer Synthesis and Sample Preparation

Poly(lactic acid) (PLA, 4042D, NatureWorks LCC, Minnetonka, MN, USA) pellets were dried in an oven at 70 °C for at least 8 hours in order to remove absorbed water from the environment. Following drying, a 0.04 g/ml PLA solution in toluene (No. 244511, Sigma-Aldrich, St. Louis, MO, USA) was prepared in an air-cooled reflux setup at 100 °C for 1.5 hours. The graft-polymers were synthetized by adding maleic anhydrite (MAh, No. 63200, Sigma-Aldrich, St. Louis, MO, USA) and 3 wt% Azobisisobutyronitrile (AIBN, No. 441090, Sigma-Aldrich, St. Louis, MO, USA), as free radical initiator (Figure 1). The PLA-MAh solution was kept at 100 °C for one hour in order to guarantee the complete decomposition of the free radical initiator. Table 1 lists the composition of the blends of PLA and MAh used in this study.

Once the polymer solution was prepared and brought to 50 °C, approximately 3 ml was poured onto pre-heated microscope glass slides (No. 12-550-A3, Fisher brand, Pittsburgh, PA, USA), 25.4 mm × 76.2 mm, and 1 mm of thickness, and maintained at 50 °C until the complete evaporation of the solvent was achieved. The films casted by these means were used for characterization purposes following the methods described below.

### 2.2. Gel Permeation Chromatography (GPC)

Molecular weight and molecular weight distribution of PLA-*g*-SAh were evaluated using gel permeation chromatography (GPC). The GPC instrument was equipped with a mixed bed column (T6000M, 300 × 8 mm, 10 μm, Malvern Panalytical, Malvern, UK), a dual detector (Viscotek GPC 270 Max, Malvern Panalytical, Malvern, UK), and a refractive index detector (Viscotek VE 3580, Malvern Panalytical, Malvern, UK). The molecular weight of the samples was obtained from calibration curves using 99 kDa polystyrene and 235 kDa polystyrene for verification. Tetrahydrofuran (THF) was used as eluent at a constant flow of 0.5 mL/min. The PLA-*g*-SAh samples were dissolved using tetrahydrofuran (THF, sample concentration of 3 mg/mL), and filtered before injection using a 0.2 µm filter.

### 2.3. Fourier-Transform Infrared Spectroscopy (FTIR)

The FTIR spectra of the polymer samples were recorded using a Fourier-transform infrared spectrometer (Agilent Cary 600 Series FTIR Spectrometer, Agilent Technologies Inc., Santa Clara, CA, USA) instrument equipped with a universal attenuated total reflectance (UATR) accessory. The spectra were recorded between a frequency range of 4000 cm^−1^ and 400 cm^−1^, and 3200 cm^−1^ and 2700 cm^−1^. 

### 2.4. Thermal Characterization

Thermal analysis was performed using a differential scanning calorimeter (DSC Model 1, Mettler Toledo, Columbus, OH, USA), under a nitrogen flow of 20 mL min^−1^. Typical sample mass was 15 mg. Analysis was performed in open Al pans. Samples were initially equilibrated at −10 °C for 5 minutes, heated from −10 °C to 210 °C at 10 °C/min, held at a temperature of 210 °C for 5 minutes, and then cooled from 210 °C to −10 °C, at a cooling rate of 10 °C min^−1^. From the DSC heating scans, glass transition temperature (T_g_), melting temperature (T_m_), and enthalpy of fusion (ΔH_m_) were determined.

The thermal stability of the graft-polymers was studied by using a thermogravimetric analyzer (TGA/DSC Model 1, Mettler Toledo, Columbus, OH, USA), equipped with the ultra-micro balance cell and differential thermal analysis (DTA) sensors, set at a heating rate of 10 °C/min under 20 mL min^−1^ nitrogen atmosphere, and a heating cycle from 25 °C to 380 °C. The thermal stability of the polymer films was defined as the onset temperature of the thermal degradation process (Td) in TGA, extracted from the Mettler Toledo STARe thermal analysis software (Mettler Toledo, Columbus, OH, USA), by taking the intercept between lines drawn tangent to the flat portion of the curve where the weight changes very little and the steep portion of the curve once the mass begins to drop. 

To enable the estimation of the degree of grafting, additional reference samples were prepared and analyzed using the same TGA method. These include: neat PLA, neat MAh, PLA-MAh50 (a 50/50 blend of PLA and MAh dissolved in toluene and cast without initiator), and PLA-AIBN3 (PLA dissolved in toluene with 3 wt% AIBN and cast). 

### 2.5. Hydrogen Nuclear Magnetic Resonance (^1^H-NMR)

Hydrogen nuclear magnetic resonance (^1^H-NMR) spectra were obtained in a Nanalysis 60 MHz NMReady-60 spectrometer (Nanalysis Corp., Calgary, AB, Canada). The equipment was pre-calibrated with deuterated toluene (No. 434388, Sigma-Aldrich, St. Louis, MO, USA). For the measurement, 10 mg of the polymer sample were dissolved in 1 mL deuterated toluene and placed in NMR tubes. Data was collected from 0 to 12 ppm with 512 scans per sample and 4096 points were recorded per scan. An analysis of the ratios of the peaks associated with MAh, PLA, and SAh was used to estimate the degree of grafting. The ^1^H-NMR spectra are included in the Supplementary Information of this manuscript.

### 2.6. Amine Response

The response of the PLA-*g*-SAh graft-polymer was studied by exposing the polymer films (2.5 cm × 7.6 cm) to the vapors from a 400 ppm methylamine (No. 426466, Sigma-Aldrich, St. Louis, MO, USA) solution in water contained in a closed cylindrical glass container (25 cm diameter × 8 cm tall) containing 300 mL of amine solution at 20 °C for 8 hours. The samples were then placed on a stainless steel platform inside the glass container such that they were suspended about 20 mm above the level of the amine solution. The reaction between the SAh-grafted polymer and methylamine, shown in Figure 1, was characterized by using FTIR techniques as per described in Section 2.3 with a scanning range from 1300 cm^−1^ and 980 cm^−1^. In addition, X-ray photoelectron spectroscopy (XPS) survey scans were performed using an X-ray photoelectron spectrometer (Kratos AXIS 165, Kratos Analytical Ltd, Manchester, UK) equipped with dual magnesium (Mg, Kα radiation hv = 1253.6 eV) and aluminum (Al, Kα radiation hv = 1486.6 eV Al), and monochromatic Al X-ray sources. XPS was performed in order to quantify the content of methylamine on the surface of the PLA-g-SAh samples after exposure to amines, as well as to collect further evidence of the reaction between them and SAh. The XPS scans were analyzed using an XPS processing software (CASAXPS 2.3.19PR1.0, Casa Software Ltd, Teignmouth, UK). Finally, the changes in thermal properties of the PLA-*g*-SAh after exposure to methylamine were measured using DSC as described in Section 2.4. In addition, a set of specimens was then exposed to water vapor (without methylamine) in order to check for selectivity of the polymer response.

### 2.7. Statistical Analysis

To test for statistically significant differences within groups of data, an analysis of variance, ANOVA with Fisher’s least significant difference (LSD) post-hoc test, was performed (using STATISTICA for Academia, StatSoft Inc., Tulsa, OK, USA) and *p*-values < 0.05 were considered statistically significant. To compare differences in properties within a certain sample type before and after exposure to amines, *t*-test analysis was performed using commercial software (STATISTICA for Academia, StatSoft Inc., Tulsa, OK, USA). *P*-values < 0.05 were considered statistically significant.

## 3. Results and Discussion

### 3.1. Molecular Weight

The molecular weight and polydispersity of both neat PLA and of the PLA-*g*-SAh samples with compositions described in Table 1 were measured by means of GPC. The results are summarized in Table 2. The molecular weight (M_w_) of the neat PLA was found to be 159 kDa ± 8 kDa, and the number average molecular weight (M_n_) and polydispersity index (PDI) were found to be 122 ± 9 kDa and 1.3 ± 0.03, respectively. For each sample, the M_w_, M_n_, and PDI was found to be statistically significantly different with respect to neat PLA. Initially, we expected to observe an increase in M_W_ after grafting corresponding to the amount of SAh grafted onto the PLA. However, for the PLA reacted with 10 wt% (PLA-*g*-SAh10 samples) and 25 wt% initial MAh (PLA-*g*-SAh25 samples), the molecular weight of each decreased significantly to 131 kDa (corresponding to an 18% decrease). Composition with initial concentrations of initial MAh of 50 wt% (PLA-*g*-SAh50) exhibited a slightly lower M_w_ than neat PLA (141 kDa ± 2 kDa). The samples that reacted with 75 wt% MAh (PLA-*g*-SAh75 samples) exhibited the highest Mw of all: 279 kDa ± 19 kDa. While the graft-polymerization, as a process of chain addition, was expected to increase the M_w_, it was found to have an opposite effect for initial MAh concentrations equal to or less than 50 wt%. As argued in previous studies, this decrease in M_w_ can be due to chain scission of the polymer chains caused by the free radicals in the polymer solution. This chain scission occurs when AIBN radicals undergo side reactions with ester groups in PLA that cause the sectioning of PLA chains [34,36,37]. For instance, Detyothin et al. [35] reported a significant decrease in the molecular weight of PLA, from about 70 kDa to 15 kDa after it was melt copolymerized with 7 wt% MAh by means of free radical polymerization with 2,5-bis(tert-butylperoxy)22,5-dime-thylhexane (Luperox 101) [35]. While this phenomenon is likely to occur at all initial concentrations of MAh, the overall values, which generally increase with MAh content, likely reflect a combination of chain scission (which decreases M_w_) and grafting (which increases M_w_). 

Similarly, the PDI constantly increased from 1.3 ± 0.03 for neat PLA to 2.7 ± 0.12 for PLA-g-SAh75. This increase suggests that there is a higher variation in the weight of the polymer chains. The source for this variation can be due to both grafting reactions and undesirable chain scission reactions. 

### 3.2. Polymer Structure

**Figure 2:**
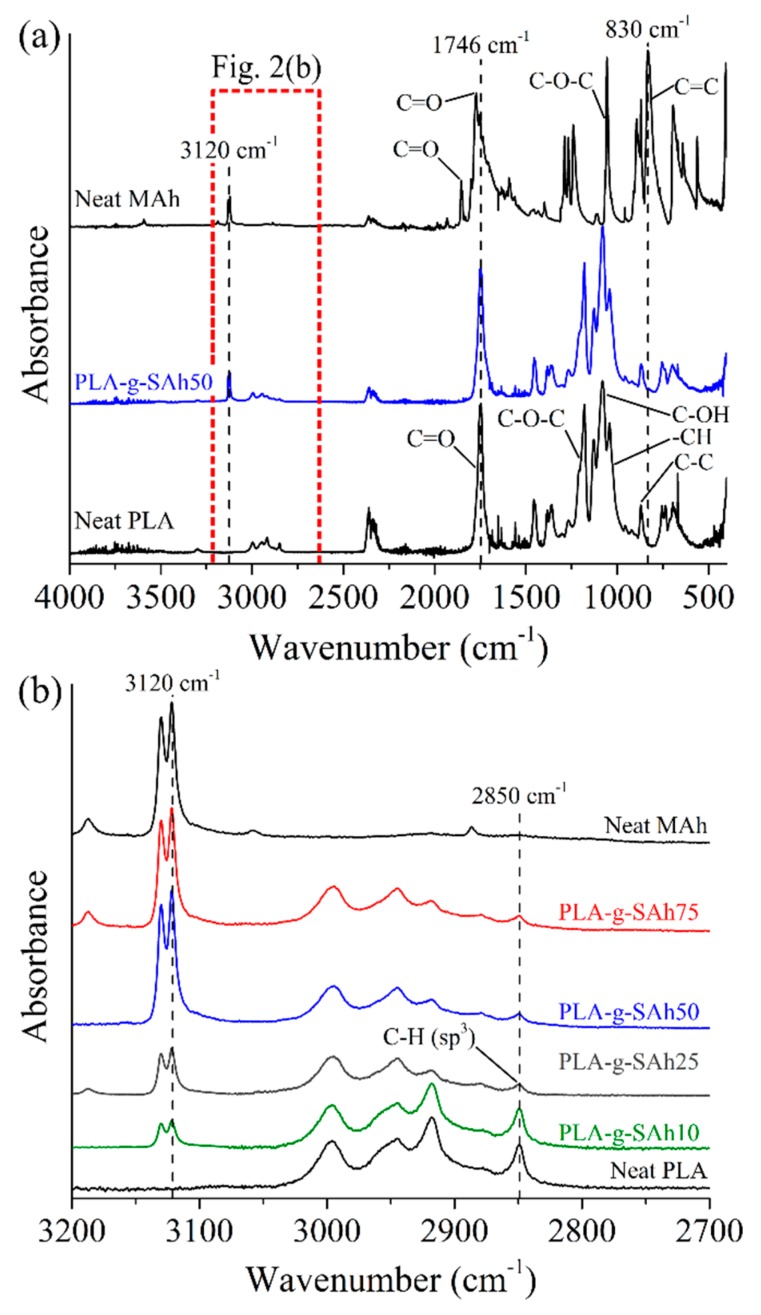
Fourier-transform infrared spectroscopy (FTIR) curves between (**a**) 4000 cm^−1^ and 400 cm^−1^, and (**b**) from 3200 cm^−1^ to 2700 cm^−1^ of neat PLA, neat MAh, PLA-*g*-SAh50, and other graft polymer samples.

The product of the polymer grafting reaction between PLA and SAh was analyzed using attenuated total reflectance ATR-FTIR. Figure 2a shows the FTIR curves for solvent cast films of neat PLA, neat MAh (powder form, as received by the provider), and PLA-*g*-SAh50 samples. Figure 2b depicts the ATR-FTIR spectra from 3200 cm^−1^ to 2700 cm^−1^ of all grafted polymers, including neat PLA and neat MAh. Neat PLA showed characteristic ATR-FTIR peaks at 1751 cm^−1^, 1182 cm^−1^, 1126 cm^−1^, 1081 cm^−1^–1035 cm^−1^, and 954 cm^−1^–872 cm^−1^, corresponding to C=O stretching, C–O–C asymmetric stretching, C–OH side group vibrations, –CH stretching, and C–C vibrations, respectively [41,42]. In the ATR-FTIR spectra of neat MAh and PLA-*g*-SAh50, the peaks at 3120 cm^−1^ (also displayed in Figure 2b) and 1781 cm^−1^ correspond to the C=O asymmetric stretching, and at 1859 cm^−1^ and 1746 cm^−1^ correspond to the C=O symmetric stretching of cyclic anhydride [28,43,44,45,46]. The presence of these asymmetric and symmetric C=O stretching bands suggest the presence of SAh in the PLA-*g*-SAh50 and other grafted polymer films. Similar to this work, Detyothin et al. monitored these symmetric and asymmetric stretches of the C=O bonds—part of the cyclic anhydride ring—to show that SAh molecules were grafted onto PLA backbone using free radical melt grafting [36]. In addition, the grafting of SAh to PLA, by the bond cleave of the C=C bond to C–C in MAh, to form SAh, and the formation of a free radicals in the C–H group of PLA, could be monitored by analyzing the C=C (830 cm^−1^ and 694 cm^−1^) and C–H (2850 cm^−1^) bond stretches [43,46]. In Figure 2a, the drop in the C=C bending bond peaks at 830 cm^−1^ and 694 cm^−1^ from neat MAh to PLA-g-SAh50 specimens suggest the process of bond cleave of the C=C bond in MAh to C–C in SAh upon the presence of free radicals from the C–H bond or from the AIBN free radical initiator. In addition, in Figure 2b, the decrease in the peaks in the proximity of the 2850 cm^−1^ band, corresponding to sp^3^ C–H stretch, may suggest the formation of free radicals in the C–H group of PLA [47,48]. Similar to previous reports, the use of ATR-FTIR to monitor relevant bonds involved in the grafting of SAh into PLA can provide some evidence of these reactions [28,43,44,45,46].

### 3.3. Thermal Properties and Degree of Grafting

**Figure 3:**
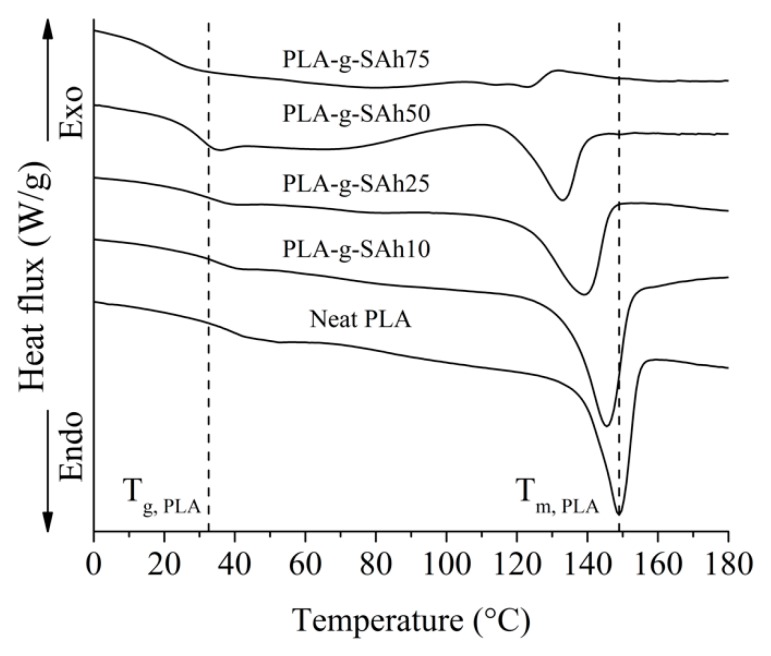
Differential scanning calorimeter (DSC) curves of neat PLA and PLA-*g*-SAh with different initial contents of MAh. Vertical lines labeled as T_g_ and T_m_ mark the glass transition and melting peak temperatures of neat PLA.

The thermal properties and thermal stability of the PLA-*g*-SAh were evaluated by means of DSC and TGA. Table 3 summarizes the glass transition temperature, T_g_, melting temperature, T_m_, and specific heat of fusion, ΔH_m_, of the neat PLA and PLA-*g*-SAh samples. In addition, the DSC curves are presented in Figure 3, where vertical lines labeled as T_g, PLA_ and T_m, PLA_ mark the glass transition temperature and the peak of the endothermic melting process of neat PLA. In general, the ΔH_m_, T_g_, and T_m_ were found to decrease with the increase of initial MAh content. For instance, the T_g_, T_m_, and ΔH_m_ decreased from to 31 °C ± 2 °C, 140 °C ± 3 °C, and 29.8 J/g ± 0.5 J/g for neat PLA to 28 °C ± 1 °C (*p* = 0.04), 116 °C ± 2 °C (*p* = 0.05), and 19.2 J/g ± 0.7 J/g (*p* = 0.001) for the PLA-*g*-SAh50 samples, respectively. As seen in Figure 3, the decrease in the thermal properties was more significant for PLA-SAh grafts with higher initial MAh content. The decrease in the polymer Mw and increase in PDI (Section 3.1) provided evidence of chain branching and chain scission during the polymer grafting reaction between PLA and SAh. This branching and scission could be responsible of the reduction of T_g_. In addition, the increase in the PDI may lead to the formation of irregular structures and amorphous regions in the polymer, further decreasing the temperature and energy required for the melting of the graft-polymer (i.e., T_m_, and ΔH_m_ [31,32,35,37,49]). The results presented in this work align with what has been reported by others [32,37]. For instance, Ma et al. [37] investigated the effects of different concentrations of MAh in a poly(lactic acid) (PLA)-*g*-maleic anhydride (MA)-co-styrene (St) copolymer that was synthetized by free radical polymerization using dicumyl peroxide (DCP) as an initiator. In their work, they reported that after copolymerization the melting point of neat PLA went from 179 °C to 176 °C for a 4.5 wt% MAh and 0.5 wt% DCP set of samples [37]. As suggested by the results in this work, and as reported by Ma et al. [37], the free radical polymerization between PLA and MAh lead to a decrease of T_g_, T_m_, and ΔH_m_ of PLA.

**Figure 4:**
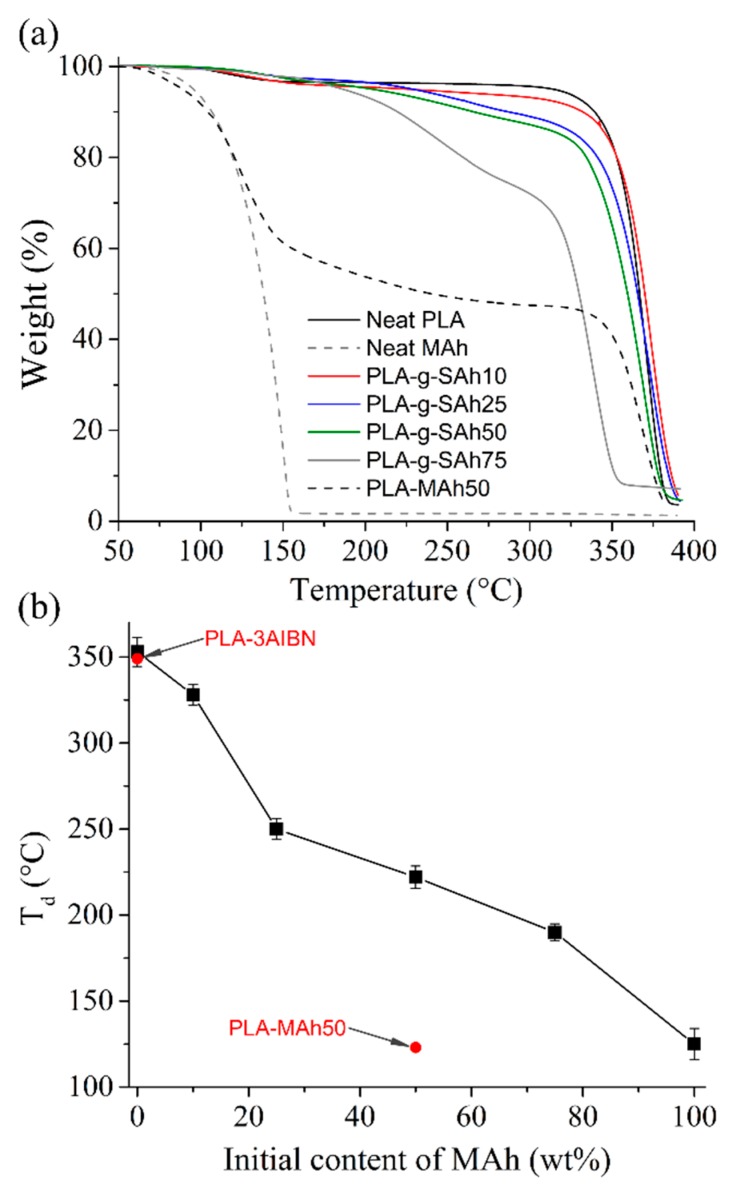
Thermogravimetric analyzer (TGA) (**a**) curves of neat PLA, neat MAh, a film comprised of a 50/50 blend of PLA and MAh without initiator (PLA-MAh50), and grafted polymers (*n* = 3); and (**b**) degradation temperature of PLA-*g*-SAh with different initial contents of MAh. Reference samples (PLA with initiator without MAh and PLA-MAh50) are denoted in red.

The thermal stability of the graft-polymer was characterized using TGA technique and defined as the onset temperature during volatilization of the specimens. Figure 4 shows representative curves of sample weight against temperature (Figure 4a) and summarizes the thermal stability properties of all compositions (Figure 4b). Neat PLA was found to be thermally stable at temperatures as high as 352 °C (T_d_). In contrast, the degradation temperature, T_d_, of neat MAh samples was found to be 120 °C. The TGA results show that the thermal stability of the graft-polymers decreased with the addition of initial MAh into the polymer solution. This degradation process was found to take place in two stages. An initial reduction of mass was found at temperatures between that of the degradation of neat MAh (120 °C) and that of neat PLA (352 °C). This is likely due to neat (unreacted) MAh molecules that became volatile at a lower temperature than neat PLA and PLA-*g*-SAh. Upon further heating, the degradation of neat PLA and PLA-g-SAh occurred. Given that this stage is more representative of the actual thermal stability of the grafted polymer, Figure 4b summarizes the onset temperature at which this second process starts. As it has been reported previously, the thermal degradation of neat PLA is associated with hydrolysis of ester groups and accelerated by the end groups (–COOH) [49,50]. While PLA-*g*-SAh graft-polymers can be expected to share the degradation mechanism of neat PLA [49], the onset of thermal degradation at lower temperatures for samples with MAh of 75 wt% may additionally be attributed in part to a wider distribution of the chain size, as evidenced by the higher PDI values for these materials (Section 3.1). In addition, Figure 4b shows the degradation temperature of the PLA-MAh50 and PLA-3AIBN specimens descried above (Section 2). No significant difference between neat PLA and PLA-3AIBN was found, suggesting that the processing method alone does not lead to the degradation of PLA. The degradation of PLA-MAh is discussed in more detail below.

While the graft-polymerization of PLA and MAh by means of free radical polymerization has been reported by Hwang et al. [32], Detyothin et al. [35,36], Ma et al. [37], Du et al. [38], Csikós et al. [39], Birnin-Yauri et al. [40], and others, the vast majority of these works use melt-based methods for the blending of polymers and creation of free radicals required for the formation of PLA-*g*-SAh. Given that these melt-based techniques are done at temperatures from 160 °C to 190 °C, it is likely that they lead to the partial or complete degradation of MAh. While these high temperatures are required for the melting and blend of PLA, they are higher than the degradation temperature of MAh, here reported to be 120 °C. In contrast in our work we report a procedure for the grafting of PLA and SAh that is based in a solution method that is executed at a temperature of 100 °C, which is well below the degradation temperature of MAh. 

The degree of grafting of SAh in the PLA polymer chains was estimated based on the results of the TGA and makes use of the fact that the T_d_ of neat MAh (120 °C) is much lower than that of neat PLA (352 °C) (Figure 4a). For each sample, the drop in mass during heating from room temperature to T_d_ of the grafted polymer was assumed to correspond to volatilization of unreacted MAh. The mass of grafted SAh was then assumed to be the difference between the initial fraction of MAh added and the fraction of mass volatilized below 120 °C. The main assumptions in this analysis are: i) all MAh initially added to the solution are incorporated into the cast polymer films; ii) all unreacted MAh is evaporated below T_d_ of the grafted polymer, and iii) PLA itself is not degraded below the T_d_ of the grafted polymer. 

**Figure 5:**
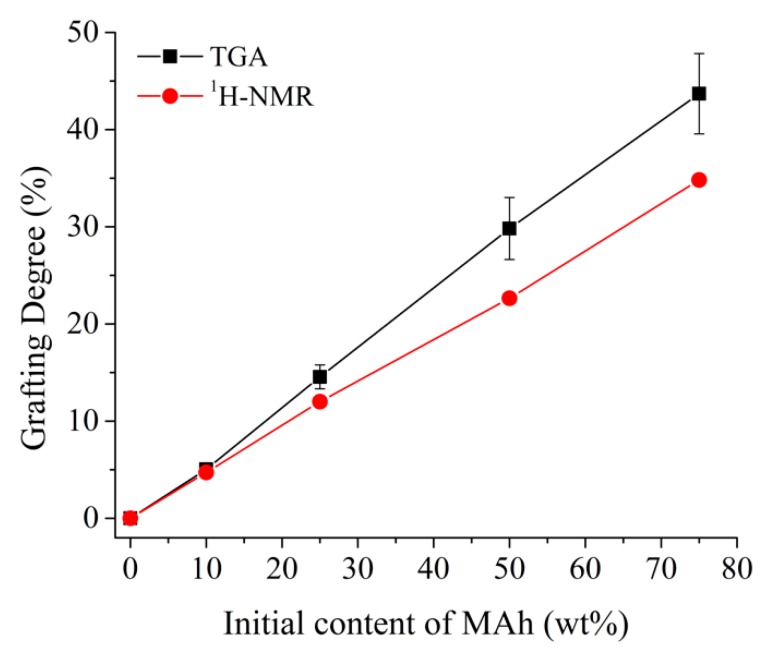
Grafting degree of grafting by TGA (*n* =3) and hydrogen nuclear magnetic resonance (^1^H-NMR) of succinic anhydride on PLA as function of the initial content of maleic anhydride concentration in solution.

All grafted samples undergo higher losses in mass below 352 °C than neat PLA: this thermal degradation is attributed to the degradation of unreacted MAh. To evaluate this assumption, samples of 50 wt% MAh (PLA-MAh50) were also characterized with TGA. The thermal degradation process of the PLA-MAh50 composition (solvent blend of PLA and MAh without free radical initiator) occurred in two stages (Figure 4a). Initially, there was a rapid drop in 50 wt% at around 120 °C, likely to be linked to the thermal degradation of neat MAh. Subsequently, the remaining 50 wt% of the samples degraded near the thermal degradation temperature of PLA. This process differs from the thermal degradation of the PLA-g-SAh50 specimens, where the initial drop of mass was 22 wt% and the remaining 78 wt% degraded at around 225 °C. The estimation of the degree of grafting assumes that the initial drop in mass (22 wt%) is linked to the degradation of unreacted MAh and that the remaining 28 wt% from the initial content of MAh (50 wt%) was grafted onto the PLA backbone. Following this rationale, the degree of grafting was estimated to be 28 wt% ± 2 wt% for the PLA-g-SAh50 composition. Figure 5 shows the estimated degree of grafting in terms of the initial content of MAh in the solution. In general, the increase in initial concentration of MAh led to a linear increase in the degree of grafting in PLA. The efficiency of grafting, calculated as the ratio between the grafting degree and initial content of MAh, was found to be 56.6% ± 3.6% for all initial contents of MAh. ^1^H-NMR spectroscopy was used to validate these results. The efficiency was found to be 46.73% ± 1.1%. The results found in both methods suggest an agreement between TGA and ^1^H-NMR methods, particularly for low initial contents of MAh (Figure 5). Similar results have been reported by Ma et al. [37]. In their work, the degree of grafting and grafting efficiency was estimated for different concentrations of MAh in a poly(lactic acid) (PLA)-*g*-maleic anhydride (MAh)-co-styrene (St) copolymer, synthetized by free radical polymerization through extrusion process and using dicumyl peroxide (DCP) as an initiator. In their work, the relation between initial MAh and grafted SAh was found to be linear (i.e., the increase in initial content of SAh led to a linear increase in grafted SAh, similar to that displayed in Figure 5). Moreover, their work reported a grafting efficiently of about 25%. The higher grafting efficiency found in the current work could be related to the extended time of reaction used (1 h), and lower processing temperature, which, as discussed above, may reduce the degradation of MAh during the grafting process. This finding suggests that the methods presented in this work have potential to provide higher grafting degree than those by melt-mixing methods. This may be particularly beneficial in applications where high grafting degree of SAh is needed, in particular those where SAh works as compatibilizer in polymer blends [35,36] and composites [49], or as a responsive element [51].

### 3.4. Amine Response: Polymer Structure

**Figure 6:**
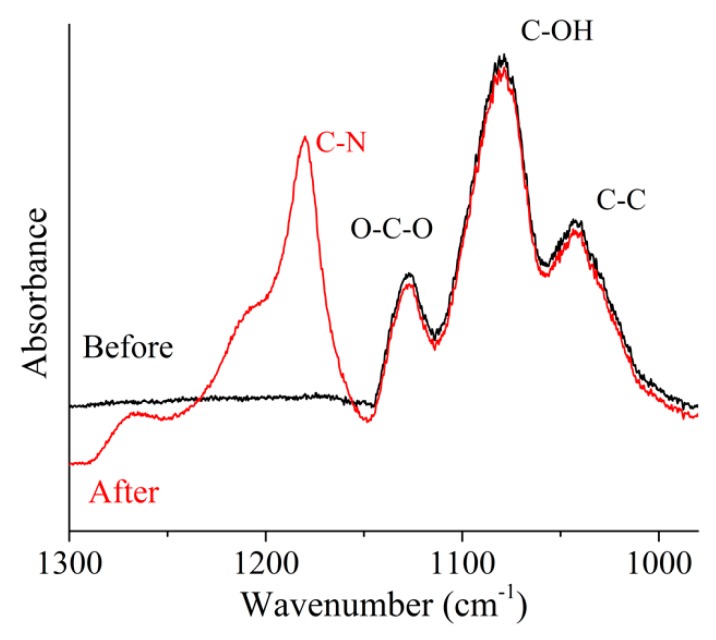
FTIR curves between 1300 cm^−1^ and 980 cm^−1^ PLA-*g*-SAh50 samples before and after exposure to methylamine vapor from a 400 ppm solution.

The reaction between SAh in the PLA-*g*-SAh and methylamine was characterized by using ATR-FTIR. The comparison of FTIR spectra between 1400 cm^−1^ and 980 cm^−1^ of PLA-*g*-SAh50 samples before and after methylamine exposure is presented in Figure 6. The spectra before exposure shows the C–O–C asymmetric stretching, C–OH side group vibrations, –CH stretching of PLA in the 1182 cm^−1^, 1126 cm^−1^, 1081 cm^−1^ and 1035 cm^−1^ bands, as previously shown in Figure 2 (Section 3.2). In addition to the PLA peaks, the spectrum (Figure 6) shows the presence of C–N stretching vibration of aliphatic amines in the 1290–1150 cm^−1^ region for the PLA-*g*-SAh films that were exposed to methylamine; this supports the reaction of SAh and methylamine proposed in Figure 1 [52].

**Figure 7:**
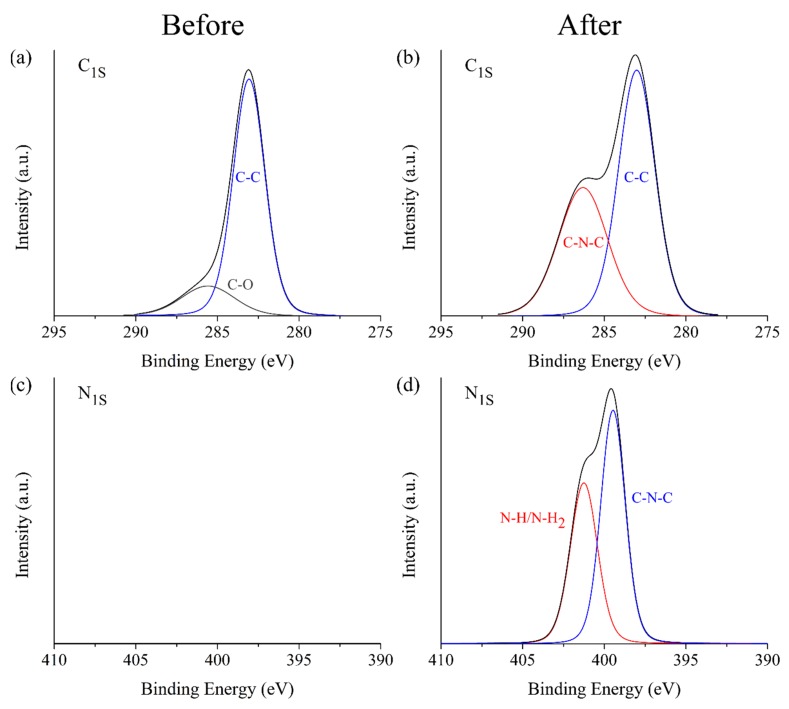
X-ray photoelectron spectroscopy (XPS) curves for (**a**,**b**) C_1S_ and (**c**,**d**) N_1S_ before and after exposure to vapors of 400 ppm methylamine solution.

XPS scans were done in PLA-g-SAh50 samples order to trace the presence of amine (e.g., –NH_2_) groups on the surface of the polymer films after exposure to methylamide vapors. As expected, carbon (C_1S_) and oxygen (O_1S_) were detected before and after exposure as they are the main constituents in the PLA-g-SAh polymer. However, in the samples exposed to methylamine, nitrogen (N) groups were detected. The XPS survey scan curves and their deconvolution for C_1S_ before and after exposure to amines are shown in Figure 7a and 7b, respectively. In addition, the scan peak for N_1S_ before and after exposure and its deconvolution after amine exposure is shown in Figure 7c and 7d. As reported previously, the C_1S_ spectrum can be deconvolved into two peak components at 285.8 eV and 283.6 eV before exposure (Figure 7a), which correspond to the carbon atoms of C–O and C–C groups, respectively. Similarly, the C_1S_ spectrum following exposure can be deconvolved into peaks at 286.9 eV and 283.6 eV, which correspond to C–N–C and C–C groups [53,54]. In addition to these two peaks in Figure 7b, those corresponding to C=O and C–O (288.3 eV and 285.8 eV) may be present but cannot be distinguished. The N_1S_ spectra (Figure 7d) can be deconvolved into two components at 401.2 eV and 399.5 eV, which correspond to the nitrogen atoms of C–N–C and N–H/N–H_2_ groups, respectively [53,54,55]. As shown in Figure 7c, there was not presence of this peak prior exposure to amines. The C–N–C peak present in both the C_1S_ and N_1S_, as well as the FTIR results discussed above, suggest that there is a reaction between the SAh group in the PLA-g-SAh and methylamine. 

### 3.5. Amine Response: Thermal Properties

**Figure 8:**
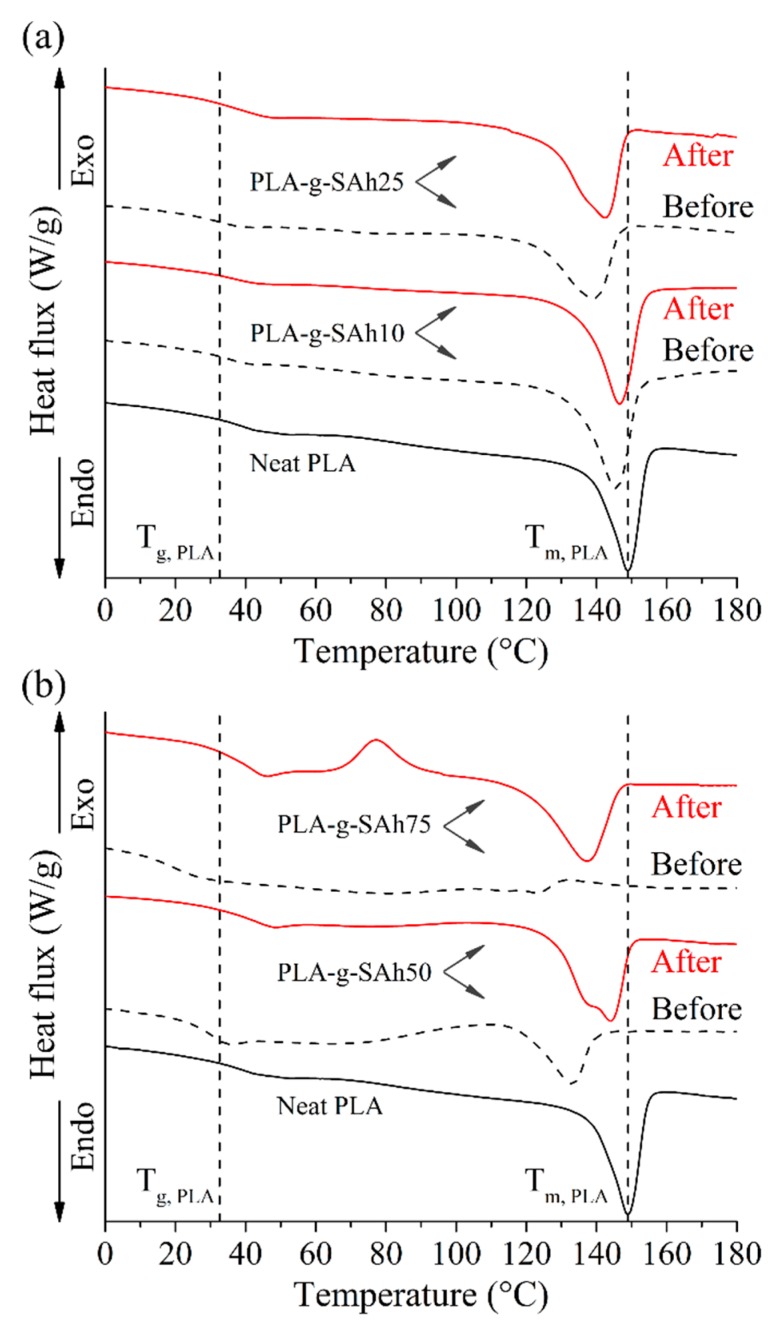
DSC curves of neat PLA and PLA-*g*-SAh with different initial contents of MAh before and after exposure to methylamine. Vertical lines labeled as T_g_ and T_m_ mark the glass transition and melting peak temperatures of neat PLA.

The thermal properties of the PLA-*g*-SAh polymer films were evaluated by DSC. Figure 8 displays the DSC curves for the graft-polymers, both before (dotted lines) and after (solid lines) exposure to methylamine. In addition, Table 4 summarizes the thermal properties from these curves. As described and argued in Section 3.3 as the initial MAh content to graft the PLA-*g*-SAh polymer increases, there is a decrease in ΔH_m_, T_g_, and T_m_ due to the chain scission that occurs during the grafting reaction between PLA and MAh. However, the exposure to methylamine was found to lead to changes in thermal properties of the graft-polymers. In general, the ring opening reaction lead to an increase in ΔH_m_, T_g_ and T_m_ in the graft-polymers when compared with the thermal properties before amine exposure. This increase was found to be more significant with increasing in MAh content. For instance, the PLA-*g*-SAh10 composition did not present a significant increase in thermal properties, while the PLA-g-SAh75 has an increase of T_g_ from 21 °C ± 2 °C to 30 °C ± 3 °C (*p* = 0.01), of T_m_ from 108 °C ± 3 °C to 121 °C ± 1 °C (*p* = 0.002), and of ΔH_m_ from 2 J/g ± 0.8 J/g to 20.6 J/g ± 0.9 J/g (*p =* 0). Little data has been reported in the past about changes in thermal properties before and after amine ring opening of SAh. Based on the results described here, we suggest that this change could be led by alterations in inter- and intra- molecular interactions in the graft-polymer. In particular, the ring opening amidation reaction of SAh to form PLA-*g*-succinamic acid (Figure 1b) and subsequent reaction with the amine group in methylamine can cause higher inter molecular interactions as a section of the polymer (SAh) opens up which can be responsible for an increase in heat and temperature required for endothermic processes such as glass transition and melting [27,56,57]. In addition, and as reported by Kesim et al. [27,57], the creation of new available OH groups can cause more intermolecular H-bonded interactions, decreasing mobility of PLA-*g*-SAh chains which contribute to that increase in thermal properties [56]. In addition, the study of thermal properties due to similar structural changes (e.g., the transition from linear to cyclic structures and vice versa), were studied by Sugai et al. [58]. In their work, the structural and thermal properties of photo-cleavable linear and cyclic poly(L-lactide) (PLLA) and poly(D-lactide) (PDLA) were studied. Similar to this work, Sugai et al. [58] reported that the transformation from a cyclic to a linear molecular structure led to an increase from 128 °C to 142 °C. This difference could be due to the higher level of physical entanglement and intermolecular interactions between polymer chains.

To further investigate the selectivity and mechanism of the response, various control samples were also characterized. A set of samples was exposed to water vapor (without methylamine), no change in the thermal properties was found. This suggests that, as illustrated in Figure 1b, water would not go through a similar reaction as that between the methylamine and SAh group in the PLA-*g*-SAh polymer converts the succinic anhydride (SAh) into the corresponding PLA-*g*-succinamic acid. In addition, to generate a blank set of samples for the reaction between SAh and methylamine into succinamic acid (succinic acid-methylamine), samples of pure PLA were exposed to both 400 ppm methylamine and water for as long 8 hours. Similarly, this did not show evidence of structural changes in PLA, perhaps because there was not SAh present to react with methylamine. In our future work, we will vary the concentration of amine in the solution to determine the limit of detection. 

## 4. Comparison with Literature

When compared with other polymers that respond to amines vapor proposed in the last three years, the grafted polymers presented in this work showed some advantages and disadvantages. Some details of these responsive polymers are summarized in Table 5. While these materials have been shown to respond to amines using different mechanisms, they require the use of additional instruments and equipment such as UV lamps in He et al. [59], quartz crystal microbalance (QCM) in Das et al. [60], and 4-point probe equipment in Khan et al. [61]. Based on the characterization done in this work, the response of change in polymer structure and thermal properties have the potential to engineer sensing elements that respond to the presence of amines by changing in color (as the ring opening reaction of SAh can protonate a pH responsive dye), which are of much interest due to their simplicity, similar to those presented by Jin et al. [14]. In contrast with recent reports, this work presents a simple solution based method for the grating of SAh onto PLA polymers chains. This method allows for a high efficiency of grafting and the potential to tailor the responsive properties of the grafted polymer with the change in initial concertation of MAh.

In contrast with the work presented by Jin et al. [14] and Khan et al. [61], the ring opening amidation reaction presented in our work is not reversible; the reverse reaction can only occur in the presence of heat or an acid. The resulting responsive material can therefore not be used for real-time monitoring of amine concentration. This irreversible behavior is, however, desirable in some applications, analogous to temperature time indicators (TTIs) where exposure to the stimulus leads to a gradual change in property—often color—of the sensor. A record of exposure to the stimulus is therefore preserved, even once the stimulus is removed.

## 5. Conclusions

In this study, we presented a solution-based method to graft PLA with SAh with a high degree of efficiency and demonstrated the response of PLA-*g*-SAh to amines. Most previous work on PLA-*g*-SAh has focused on using melt-mixing; one advantage of our technique is that it takes place well below the degradation temperature of MAh/SAh. In addition, we achieved a higher efficiency of grafting than achieved previously by melt mixing (e.g., Ma et al. [37] and Hwang et al. [32]); this allowed for a high degree of grafting overall. The structural and thermal properties of the graft-polymers were studied. The increase in branching in the PLA chains, as consequence of grafting, led to a decrease in melting point and crystallinity of the polymer (as compared with neat PLA). Following grafting, PLA-*g*-SAh films were exposed to the vapor of a 400 pm amine solution. This concentration was selected as it is indicative of spoilage in fresh fish products [4]. While other studies have reported the reaction between SAh-grafted polymers (such as poly(methyl vinyl ether-co-maleic anhydride) (PMVE-*co*-MAh) [63]) and amines, the study of the effect of the degree of grafting on the reaction with amines has not been explored in the past. Our materials exhibited significant changes in thermal properties after exposure to the amine vapor, as the methylamine caused ring opening of SAh, increased physical interactions and OH–bonded interactions of the polymer which increased its thermal properties. Due to the structural changes that occur within the grafted polymer upon exposure to the amine vapor, this work presents potential opportunities for the development of real-time amine sensing elements that are simple to use, biodegradable, and simple to produce at a large scale. In our future work we will leverage the reaction that occurs between the amine vapor and SAh groups to engineer colorimetric sensors.

## Figures and Tables

**Table 1:** Sample composition.

Sample Name	Poly(lactic acid) (PLA) *Wt %*	Maleic anhydrite (MAh) *Wt %*	Azobisisobutyronitrile (AIBN) *Wt %*
Neat PLA	100	0	0
PLA-*g*-SAh10	87	10	3
PLA-*g*-SAh25	72	25	3
PLA-*g*-SAh50	47	50	3
PLA-*g*-SAh75	22	75	3

**Table 2:** Summary of gel permeation chromatography (GPC) results (*n* = 3).

Sample Name	Molecular number (M_n_) *kDa*	Molecular weight (M_w_) *kDa*	Polydispersity index (PDI) *M_w_/M_n_*
Neat PLA	122 ± 9	159 ± 8	1.3 ± 0.03
PLA-*g*-SAh10	80 ± 4	131 ± 4	1.6 ± 0.09
PLA-*g*-SAh25	81 ± 6	131 ± 2	1.6 ± 0.09
PLA-*g*-SAh50	85 ± 1	141 ± 2	1.7 ± 0.03
PLA-*g*-SAh75	102 ± 2	271 ± 19	2.7 ± 0.12

**Table 3:** Thermal properties from DSC of neat PLA and PLA-g-SAh (*n* = 3, *p* < 0.01).

Sample Name	T_g_ (°C)	T_m_ (°C)	ΔH_m_ (J/g)
Neat PLA	31 ± 2	140 ± 3	29.8 ± 0.5
PLA-g-SAh10	32 ± 1	133 ± 2	21.8 ± 0.4
PLA-g-SAh25	30 ± 2	121 ± 3	20.6 ± 0.6
PLA-g-SAh50	28 ± 1	116 ± 2	19.2 ± 0.7
PLA-g-SAh75	21 ± 2	108 ± 3	2 ± 0.8
MAh	-	53	138

**Table 4:** Thermal properties from DSC of neat PLA and PLA-*g*-SAh before and after exposure to methylamine (*n* = 3).

	Before			After		
Sample Name	T_g_ (°C)	T_m_ (°C)	ΔH_m_ (J/g)	T_g_ (°C)	T_m_ (°C)	ΔH_m_ (J/g)
PLA-*g*-SAh10	32 ± 1	133 ± 2	21.8 ± 0.4	31 ± 3	142 ± 2	24.8 ± 0.4
PLA-*g*-SAh25	30 ± 2	121 ± 3	20.6 ± 0.6	32 ± 2	135 ± 1	26.9 ± 0.6
PLA-*g*-SAh50	28 ± 1	116 ± 2	19.2 ± 0.7	31 ± 1	126 ± 2	23.8 ± 0.5
PLA-*g*-SAh75	21 ± 2	108 ± 3	2 ± 0.8	30 ± 3	121 ± 1	20.6 ± 0.9

**Table 5:** Relevant responsive polymers that react to amines (from 2017 to 2019).

Material	Amines	Mechanism	Response	Ref.
Nitrated polythiophene (NPTh)	Ethylenediamine, putrescine, cadaverine, spermidine, phenethylamine, histamine	The biogenic amine (BA) easily diffuses into the polymer film and forms charge transfer complexes with NPTh. These NPThδ+-BAδ− complexes lead to the change in color of the film.	A fast change in color from light brown to a highly deep dark brown	[14]
Alkaline earth metal–organic coordination polymer	Methylamine, dimethylamine, trimethylamine	Amines combine with unsaturated carboxylic groups in the polymer. The carboxylic group can no longer vibrate, increasing the rigidity and educe the loss of non-radiation energy of the ligand, causing the increase of the fluorescence emission intensity	On/off change in fluorescence with initial and final peaks at 525 nm and 612 nm	[59]
Polyaniline-titanium(IV) sulphosalicylophosphate composite	Methylamine, ethylamine	The lone pair of nitrogen of amine interacts with the imine nitrogen of polyaniline, decreasing the intensity of positive charge which decreases the conductivity	Reversible change in resistivity measured with 4-point probe	[61]
Schiff base 3(aminopropyl)triethoxysilane (APTES)	Methylamine, ethylamine, diethylamine, triethylamine, tertbutylamine ammonia	Small molecules of amines are trapped in molecular pores introduced by the bulky group of Schiff base attached to polysiloxane. They are stabilized by H-bonds and dipole-dipole interaction	Polymer-coated quartz crystal microbalance (QCM) substrate absorbs the amines, increasing the mass of the film and the mass	[60]
EuCl3 with 4,4′-biphenyldicarboxylic acid (H2BPDC)	Methylamine, dimethylamine, trimethylamine	Fluorescence quenching by the amines	Drop in fluorescent emission at 413, 578, 592, 614 (main peak), 650 and 704 nm when excited by UV light of 311 nm	[62]
PLA-g-SAh	Methylamine	Amidation of SAh in PLA-g-SAh with methylamine. Lone electron pair of the amine conducts a nucleophilic attack on the C=O π bond of SAh to start a ring opening reaction	Increase in melting point and donation of protons during ring opening of SAh. Potential in color change indicators and electrochemical sensing	This work

## Supplementary Materials

The following are available online at https://www.mdpi.com/2073-4360/11/9/1466/s1.
