# Supplementary material for: Amine Responsive Poly(lactic acid) (PLA) and Succinic Anhydride (SAh) Graft-Polymer: Synthesis and Characterization
Source: Polymers (Basel). 2019 Sep 7;11(9):1466. doi: 10.3390/polym11091466 (PMC6780798; doi:10.3390/polym11091466)
Supplement: Supplementary file 1 [file polymers-11-01466-s001.pdf]

Article

Amine Responsive Poly(lactic acid) (PLA) and Succinic Anhydride (SAh) Graft-Polymer: Synthesis and Characterization - Supplementary Information

Adrián Lopera-Valle[1], Anastasia Elias[1,*]

[1] *Department of Chemical and Materials Engineering, University of Alberta, Donadeo Innovation Centre for Engineering, Edmonton, Alberta, T6G 1H9, Canada;* lopera@ualberta.ca (A.L-V); aelias@ualberta.ca (A.E.)
* Correspondence: aelias@ualberta.ca

Received: date; Accepted: date; Published: date

1. Proton nuclear magnetic resonance (^{1}H-NMR)

The ^{1}H-NMR spectra, Figure S1, were used to estimate the degree of grafting on SAh into PLA. The spectra for neat PLA shows the representative signals at 1.2-1.5 and 5.0-5.2 ppm corresponded to -CH^3 and -H respectively. Neat MAh shows a signal at around 7 ppm, corresponding to –H. After grafting, the signal for the succinic anhydride (SAh) group was found in the region of 2-2.2 ppm [1, 2]. The estimation of the degree of grafting by comparing the area of the peaks associated to MAh and SAh. The degree of grafting is reported in Figure 5.

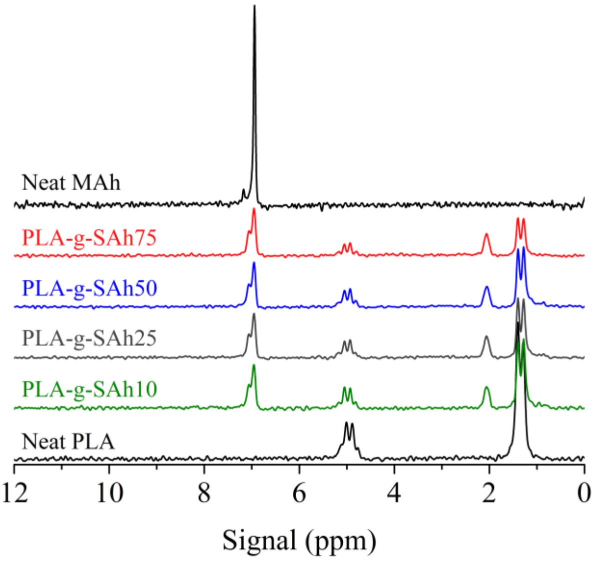

Figure S1. ^{1}H-NMR spectra of neat PLA, neat MAh, and other graft polymer samples

7. Reference

1. Khankrua, R.; Pivsa-Art, S.; Hiroyuki, H.; Suttiruengwong, S. Grafting of poly (lactic acid) with maleic anhydride using supercritical carbon dioxide, *IOP Conf. Ser. Mater. Sci. Eng.*, **2015**, *vol. 87*, *no. 1*, p. 012066. DOI: 10.1088/1757-899X/87/1/012066.
2. Hwang, S. W. *et al.* Grafting of maleic anhydride on poly(L-lactic acid). Effects on physical and mechanical properties, *Polym. Test.*, **2012**, *vol. 31*, *no. 2*, pp. 333–344. DOI: 10.1016/j.polymertesting.2011.12.005.

© 2019 by the authors. Submitted for possible open access publication under the terms and conditions of the Creative Commons Attribution (CC BY) license (http://creativecommons.org/licenses/by/4.0/).
